# Dynamic proteomic changes and ultrastructural insights into *Pochonia chlamydosporia*’s parasitism of *Parascaris equorum* eggs

**DOI:** 10.3389/fcimb.2025.1600620

**Published:** 2025-08-20

**Authors:** Luyao Hao, Fengmiao Zhao, Hongyou Liu, Chengyu Ma, Xiaoqing Jia, Lili Jiang, Zhaobin Fan, Rui Wang

**Affiliations:** ^1^ College of Veterinary Medicine, Inner Mongolia Agricultural University, Hohhot, China; ^2^ Key Laboratory of Clinical Diagnosis and Treatment of Animal Diseases, Ministry of Agriculture, National Animal Medicine Experimental Teaching Center, Hohhot, China; ^3^ College of Grassland Science, Inner Mongolia Agricultural University, Hohhot, China; ^4^ College of Pharmacy, Heze University, Heze, China

**Keywords:** helminth eggs-parasitic fungi, biological control, differential protein expression, proteomic analysis, host-pathogen interaction

## Abstract

*Pochonia chlamydosporia* (Goddard) Zare & Gams (Ascomycota, Sordariomycetes, Hypocreales, Pochoniaceae, *Pochonia*) is a nematophagous fungus with significant potential as a biocontrol agent against animal-parasitic nematodes. However, the molecular and cellular mechanisms underlying its infection process remain poorly understood.This study comprehensively investigated *P. chlamydosporia* infection dynamics in *Parascaris equorum* eggs using both microscopic and proteomic approaches. Infection was monitored at three distinct stages (early, middle, and late). Microscopic analysis included scanning electron microscopy (SEM), transmission electron microscopy (TEM), and light microscopy (LM) to observe morphological changes. A 4D-DIA-based quantitative proteomics approach was employed to analyze exoproteomic changes, and quantitative PCR (qPCR) was used to validate key genes. Microscopic observations revealed progressive invasion of *P. chlamydosporia* into nematode eggs, with detailed morphological changes in both fungal structures and eggs, including key stages such as attachment, germination, and egg degradation. Proteomic analysis identified 410 differentially expressed proteins (DEPs) across the three stages, with 313 upregulated and 403 downregulated. Gene Ontology (GO) enrichment analysis showed DEPs involvement in cellular stress response, proteolysis, metabolic process, and hydrolase activity. Kyoto Encyclopedia of Genes and Genomes (KEGG) pathway analysis identified key pathways including signal transduction, cell wall biosynthesis, energy metabolism, and host-pathogen interactions. qPCR validation further supported the molecular basis of parasitic behavior. These findings clarify that *P. chlamydosporia* employs a highly coordinated molecular strategy to adapt to and exploit its host, contributing to our understanding of fungal-nematode interactions and laying a solid foundation for developing *P. chlamydosporia* as a sustainable tool for integrated pest management.

## 1 Introduction

*Pochonia chlamydosporia* is a well-recognized egg-parasitic fungus with significant potential as a biological control agent against animal-parasitic helminths, which are major pathogens causing substantial health and economic burdens worldwide (Fonseca et al., 2024; Hao et al., 2025). This fungus exhibits a dual lifestyle: saprophytic in the soil and parasitic on helminth eggs, making it an eco-friendly and sustainable tool for integrated parasitic control strategies (Manzanilla-López et al., 2013). Notably, *P. chlamydosporia* has demonstrated effectiveness in controlling parasitic nematodes such as *Ascaris suum*, *Trichuris* spp., and other economically significant helminth species (Braga et al., 2016; Lelis et al., 2014; Ferreira et al., 2011; Silva et al., 2010). In addition to its well-documented role in nematode control, *P. chlamydosporia* has also been investigated for its potential in controlling diseases transmitted by other vectors, such as snails and other mollusks (Castro et al., 2022, 2019).

The molecular mechanisms underlying *P. chlamydosporia*’s parasitism remain poorly understood despite its potential applications. While previous studies have explored its biological traits (Larriba et al., 2014), interactions with animal hosts (Avelar Monteiro et al., 2017), and production of extracellular hydrolytic enzymes that penetrate helminth eggs (Morton et al., 2003), the roles of extracellular proteins secreted during different infection stages remain largely unexplored. A comprehensive proteomic analysis of these proteins is critical, as they play essential roles in pathogen-host interactions, including host invasion, immune suppression, and environmental modification to facilitate infection. For nematophagous fungi like *P. chlamydosporia*, extracellular proteins are central to pathogenicity. Nematode eggshells, primarily composed of proteins, chitin, and lipids, depend on chitin for structural integrity (Monteiro et al., 2017). Research indicates that *P. chlamydosporia* secretes hydrolytic enzymes capable of degrading the protein and chitin layers of nematode eggs (Aranda-Martinez et al., 2016; Bezerra et al., 2021; Yang et al., 2013), increasing permeability, inflicting morphological damage on eggshells and embryos, and ultimately rendering the eggs nonviable, effectively suppressing nematode reproduction (Braga et al., 2010; Braga and de Araújo, 2014).

To build on this understanding, advances in proteomic technologies, particularly mass spectrometry-based approaches such as data-independent acquisition (DIA), provide powerful tools to comprehensively profile the extracellular proteins secreted by *P. chlamydosporia* during different stages of infection. DIA methods, especially four-dimensional DIA (4D-DIA), offer superior sensitivity, reproducibility, and proteome coverage compared to traditional proteomic techniques such as isobaric tags for relative and absolute quantitation (iTRAQ) or gel-based methods (Bruderer et al., 2015; Gillet et al., 2012). These advanced techniques have already been successfully applied in studying fungal pathogenicity and host-pathogen interactions, revealing key proteins and pathways involved in infection processes (Girard et al., 2013; Yang et al., 2010), making them well suited for investigating the molecular mechanisms underlying *P. chlamydosporia*’s parasitism.

Here, we applied a 4D-DIA-based approach to analyze extracellular proteomic changes in *P. chlamydosporia* during three infection stages (3, 5, and 7 days) of nematode eggs to identify key pathways and proteins involved in antagonism and to explore its molecular mechanisms and potential as a biocontrol agent.

## 2 Materials and methods

### 2.1 Fungal strain

The *Pochonia chlamydosporia* strain used in this study, originally isolated from Yunnan Province, China, is deposited under the accession number ACCC 30601 at the Agricultural Culture Collection of China (ACCC) and preserved at −80°C in the Parasitology Laboratory of Inner Mongolia Agricultural University, Hohhot, China.

### 2.2 Obtaining *Parascaris equorum* eggs

Collect fresh adult female *P. equorum* worms from mare feces. Wash the worms three times with warm water and temporarily store them in sterile physiological saline. Secure the worms on a wax tray, and excise and dissect the terminal portion of the uterus. Gently squeeze the uterus and flush the interior walls repeatedly with physiological saline using a syringe to collect the eggs. The final collected egg suspension was resuspended in sterile water for future use.

### 2.3 Induced mycelial culture

Three portions of LMZ liquid medium (containing 2 g·L^-1^ gelatin, 8 g·L^-1^ peptone, 1 g·L^-1^ yeast extract, 0.01 g·L^-1^ FeSO_4_·7H_2_O, and 0.5 g·L^-1^ MgSO_4_·7H_2_O) were prepared. Then, 1 ml of a pre-prepared spore suspension (1 × 10^6^ spores/ml) was added to each portion of the liquid medium. The volume of each portion was adjusted to 150 ml with LMZ liquid medium. The cultures were incubated at 26°C with shaking at 150 r/min for 3 days. After 3 days, three of the portions were each supplemented with 1 ml of *P. equorum* eggs at a final concentration of 10,000 eggs/150 ml. The fermentation filtrates were obtained by filtering the culture through sterile Whatman No. 1 filter paper to remove mycelia, followed by centrifugation at 10,000 × g for 15 min at 4°C. The supernatant was then sterile-filtered through a 0.22 μm membrane, collected at 3 (A1), 5 (B1), and 7 (C1) days, aliquoted, and stored at −80°C until use. All treatments were set with three biological replicates to ensure the reliability and reproducibility of the results. These samples were specifically reserved for subsequent proteomic sequencing analysis to investigate the dynamic changes in the proteome during the different time points of the fermentation process, which is crucial for understanding the underlying biological mechanisms and potential applications related to the fermentation system.

### 2.4 Infection process observed by microscopy

#### 2.4.1 Observation with light microscope

Mycelial plugs of *P. chlamydosporia* were transferred onto 9-cm disposable petri dishes containing 2% water agar (WA) solid medium. Cellulose dialysis membrane disks (9 cm diameter) were autoclaved at 120°C for 15 min in distilled water, then clamped onto the agar surface to cover it completely, ensuring the membrane edges adhered to the plate rim. This setup facilitated nutrient diffusion while physically separating the fungus from nematode eggs. Next, plates were incubated in the dark, at a temperature of 26°C. Five hundred eggs were evenly distributed at five distinct positions on each petri dish containing 2% water agar (WA) solid medium, with approximately 100 eggs placed at each designated spot to ensure uniform coverage. The treated group was established by co-culturing these eggs with *P. chlamydosporia* (pre-cultured for 3 days), while the control group omitted fungal inoculation.

#### 2.4.2 Observation with scanning electron microscope

After the observation of the interaction with *P. equorum* eggs, pieces of the dialysis membrane with eggs parasitized by fungi exposed to capture were cut with the aid of a blade, collected with a fine-tipped clamp, and fixed in glutaraldehyde at 2.5% in 0.05 M of phosphate buffer for 24h. Next, they were washed five times in the same buffer and dehydrated by passing the material in a series of ethyl alcohol (30%, 50%, 70%, 90%, and 100%). The material was dried in a critical point dryer BALZERS1 using carbon dioxide and recovered with gold plating. The procedure of sample treatment was performed as previously published (Hao et al., 2025; Zhang et al., 2020).

#### 2.4.3 Observation with transmission electron microscope

In this experiment, the stages of the infection process were observed by transmission electron microscope (TEM). Fresh tissue samples were quickly fixed in a cold fixative solution for 2–4 h, followed by washing with 0.1M phosphate buffer (pH 7.4). The samples were then fixed in a mixture of 1% osmium acid and 0.1M phosphate buffer (pH 7.4) for 2h at room temperature. After fixation, the samples underwent dehydration using graded ethanol and acetone series. Infiltration and embedding steps were performed to prepare the samples for observation. Ultra-thin sections were cut from the embedded samples using a diamond knife. Staining with uranium lead double staining was done to improve visibility. The sections were then observed using a transmission electron microscope to visualize the various stages of the infection process. Images were captured and analyzed for detailed study of the samples.

### 2.5 Protein preparation

#### 2.5.1 Protein extraction

Take an appropriate volume of each sample, freeze-dry it, and add a suitable amount of SDT lysis buffer (4% SDS, 100 mM Tris-HCl pH 7.6, 1 mM DTT, 1× protease inhibitor cocktail). Transfer the solution to an EP tube, perform a boiling water bath for 3 min, then ultrasonic disruption (600 W, 30% duty cycle, 10 cycles) for 2 min. Centrifuge at 16,000*g* for 20 min at 4°C, and collect the supernatant.

#### 2.5.2 Protein digestion

Each sample was treated with an appropriate amount of protein for FASP enzymatic digestion. DTT was added to a final concentration of 100 mM, followed by a boiling water bath for 5 min, then cooled to room temperature. Subsequently, 200 μl of UA buffer (8 M Urea, 150 mM Tris-HCl, pH 8.0) was added, mixed thoroughly, and transferred to a 10 kDa ultrafiltration centrifuge tube, then centrifuged at 12,000*g* for 15 min. Another 200 μl of UA buffer was added, centrifuged at 12,000*g* for 15 min, and the filtrate was discarded. Next, 100 μl of IAA solution (50 mM IAA in UA buffer) was added, shaken at 600 rpm for 1 min, incubated at room temperature in the dark for 30 min, and centrifuged at 12,000*g* for 10 min. After this, 100 μl of UA buffer was added and centrifuged at 12,000*g* for 10 min, a process repeated twice. Then, 100 μl of NH_4_HCO_3_ buffer was added and centrifuged at 14,000*g* for 10 min, also repeated twice. Following this, 40 μl of Trypsin buffer (6 μg Trypsin in 40 μl NH_4_HCO_3_ buffer) was added, shaken at 600 rpm for 1 min, and incubated at 37°C for 16–18 h. After digestion, the collection tube was replaced, and the sample was centrifuged at 12,000*g* for 10 min to collect the filtrate, which was then mixed with an appropriate amount of 0.1% TFA solution. The digested peptides were desalted using a C18 cartridge and vacuum freeze-dried. Finally, the dried peptides were redissolved in 0.1% FA, and the peptide concentration was measured in preparation for LC-MS analysis.

### 2.6 DIA mass spectrometry data acquisition

For each sample, an appropriate amount of peptides was used for chromatographic separation on the Vanquish Neo UHPLC system (Thermo Fisher Scientific, Waltham, Massachusetts, USA). The mobile phase consisted of buffer A (0.1% formic acid in water) and buffer B (0.1% formic acid in acetonitrile, 80% acetonitrile). The column was equilibrated with 96% buffer A prior to sample injection. The sample was first injected into the Trap Column (PepMap Neo 5 μm C18, 300 μm × 5 mm, Thermo Scientific) and subsequently separated using the analytical column (μPAC Neo High Throughput column, Thermo Scientific) under the following gradient conditions: 0 min–0.1 min, linear gradient of buffer B from 4% to 6%; 0.1 min–1.1 min, linear gradient of buffer B from 6% to 12%; 1.1 min–4.3 min, linear gradient of buffer B from 12% to 22.5%; 4.3 min–6.1 min, linear gradient of buffer B from 22.5% to 45%; 6.1 min–8 min, buffer B maintained at 99%. After separation, the peptides were analyzed using an Orbitrap Astral mass spectrometer (Thermo Scientific) with DIA mode. The analysis duration was 8 min with the following parameters: electrospray voltage of 2.2 kV, positive ion detection mode, precursor ion scan range of 380–980 m/z, MS1 resolution of 240,000 at m/z 200, AGC target of 5e5 ions, and MS1 maximum injection time (Maximum IT) of 3 ms. MS2 resolution was set to 80,000, AGC target to 500%, and MS2 Maximum IT to 3 ms, with an RF-lens setting of 40%. MS2 activation was performed using HCD with a normalized collision energy of 25%, an isolation window of 2 Th, and a cycle time of 0.6 s.

### 2.7 DIA mass spectrometry data search

All mass spectrometry data were processed and searched using the DIA-NN software, which performed database searching and DIA-based protein quantification. The database used was uniprotkb-Metacordyceps chlamydosporia (Nematophagous fungus) (*Pochonia chlamydosporia*) [280754]-14275-20241104.fasta, obtained from (https://www.uniprot.org/taxonomy/280754).

### 2.8 Data analysis

The analysis parameters for the DIA-NN software are shown in **Table 1**.

**Table 1:** Data-independent acquisition neural network (DIA-NN) software analysis parameters.

Item	Value
Enzyme	Trypsin
Enzyme trypsin	1
Fixed modifications	Carbamidomethyl (C)
Variable modifications database	Oxidation (M), Acetyl (Protein N-term) uniprotkb-Metacordyceps chlamydosporia (Nematophagous fungus) (*Pochonia chlamydosporia*) [280754]-14275- 20241104.fasta
Database pattern	Target-Reverse
PSM (Peptide-Spectral Matching) FDR	0.01
Protein FDR	0.01

Proteins meeting the rigorous criteria of a fold change greater than 1.5 (either upregulated or downregulated) and exhibiting a *P*-value below 0.05 were designated as significantly DEPs. The method used for comparing two groups was the unpaired Student’s t-test. Multiple group comparative analysis defaulting to one-way analysis of variance (ANOVA) testing method was employed, with proteins exhibiting *P* < 0.05 selected as proteins with differential expression.

The data were analyzed and visualized using GraphPad Prism 8.0 software, and the results are presented as the means ± standard errors of the means (SEMs). The significance level was set at *p* < 0.05, and the outcomes are reported as the means ± SEMs. Three biological replicates were utilized for all experiments.

### 2.9 Bioinformatic analysis

The FASTA protein sequences of differentially changed proteins were blasted against the online Kyoto Encyclopedia of Genes and Genomes (KEGG) database (http://geneontology.org/) to retrieve their KOs and were subsequently mapped to pathways in KEGG. The corresponding KEGG pathways were extracted.

### 2.10 Real-time quantitative reverse transcription PCR

Total RNA was extracted from samples using a standard RNA extraction kit (Omega, USA), and its quality was confirmed by measuring the OD260/280 ratio (1.8–2.0). Reverse transcription was performed to synthesize cDNA from the extracted RNA following the manufacturer’s protocol (TaKaRa, Dalian, China). qRT-PCR reactions were prepared in 20 μl volumes containing 10 μl of TB Green^®^ Premix Ex Taq™ II (TaKaRa, Dalian, China), 0.4 μM of each primer, 2 μl of diluted cDNA template, and nuclease-free water, under the following conditions: 95°C for 30 s (initial denaturation), followed by 40 cycles of 95°C for 5 s (denaturation) and 60°C for 30 s (annealing/extension).

The relative expression levels of target genes were calculated using the 2^-△△CT^ method, with β-actin as internal reference genes, where ΔCT represents the difference in CT values between the target and reference genes, and △△CT represents the difference between the experimental and control groups. The data are shown as the means ± SEMs of three independent experiments.

## 3 Results

### 3.1 Infection process observed by microscopy

The infection process of *P. chlamydosporia* mycelium on egg was observed in three stages: early, middle, and late.

**Figure 1:**
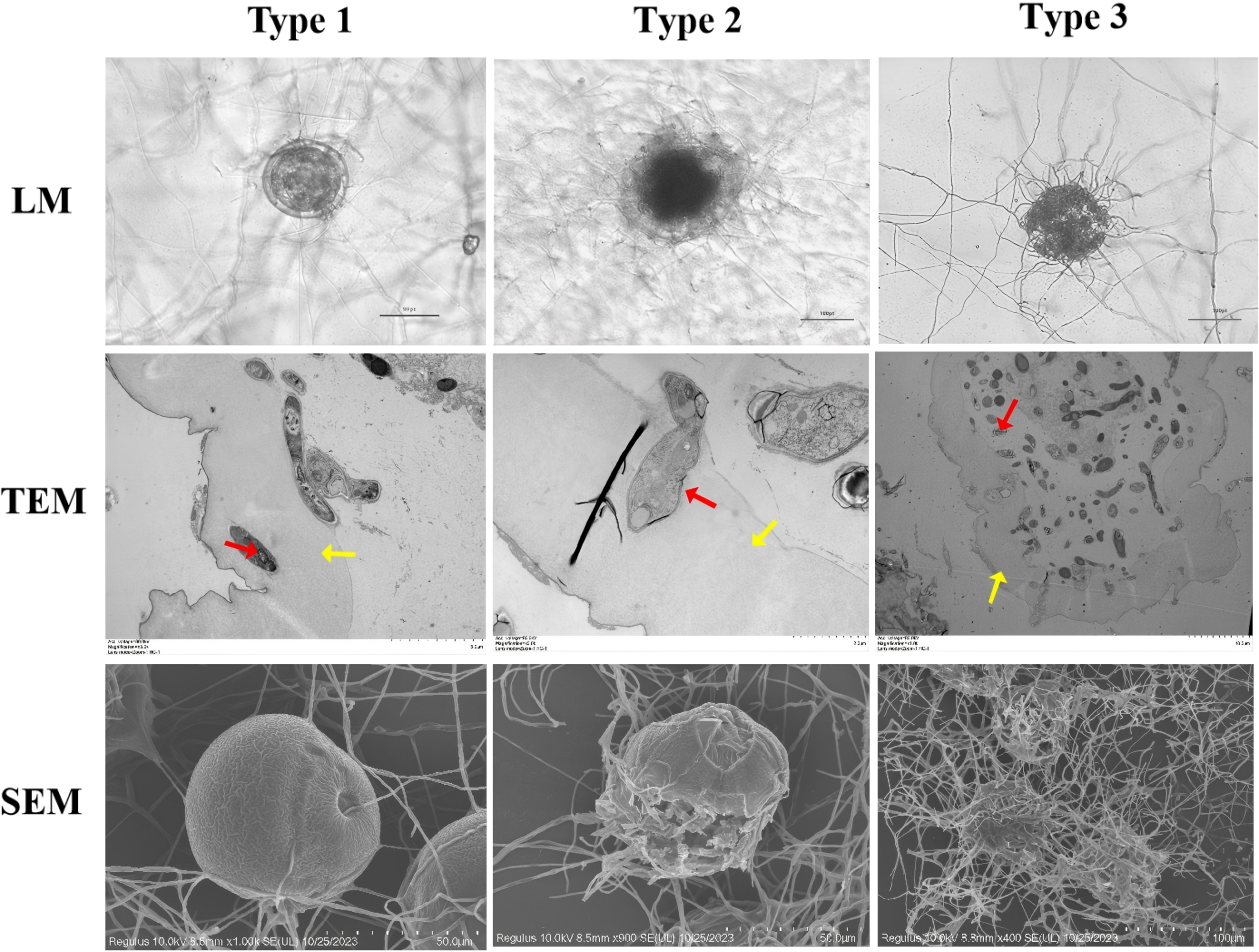
Microscopy of development and morphological structure of *Parascaris equorum* eggs. (*Pochonia chlamydosporia*: red arrow, *Parascaris equorum* Eggs: yellow arrow). Type 1 effect: physiological and biochemical impact without morphological damage to the eggshell, with visible hyphae adhering to the eggshell; Type 2 effect: lytic effect causing morphological changes in both the embryo and eggshell, without hyphal penetration; Type 3 effect: lytic effect with morphological changes in the embryo and eggshell, along with hyphal penetration and internal egg colonization.

Mycelial growth intensified within 12h, forming dense hyphal masses around the eggshell and producing conidia. By 24h, the mycelium completely covered the egg surface. SEM revealed attachment structures at hyphal tips penetrating the eggshell, while TEM showed initial hyphal penetration signs (**Figure 1**, Type 1).

During the middle stage (24–72h), the mycelium enveloped the eggshell, with TEM showing degradation of internal architecture (membrane rupture and cellular disintegration; **Figure 1**, Type 2).

In the late stage (72–120h), mycelial invasion led to near-total dissolution of the eggshell. TEM images showed complete mycelial penetration, causing severe internal damage and cellular collapse, with nearly all internal contents consumed (**Figure 1**, Type 3).

### 3.2 Overview of 4D-DIA data analysis

We conducted a rigorous screening of the experimental data samples to ensure the association of identified proteins with sample groups containing a minimum of 50% non-missing values. Subsequently, any remaining missing values were meticulously imputed prior to the statistical analysis. Our proteomic analysis generated a substantial 21,220 mass spectra. Following meticulous data filtration to exclude low-scoring spectra, we successfully matched 17,859 unique spectra to 1,859 unique peptides, resulting in the identification of a final set of 2,340 proteins. Subsequently, any remaining missing values were meticulously filled in before conducting the statistical analysis.

**Figure 2:**
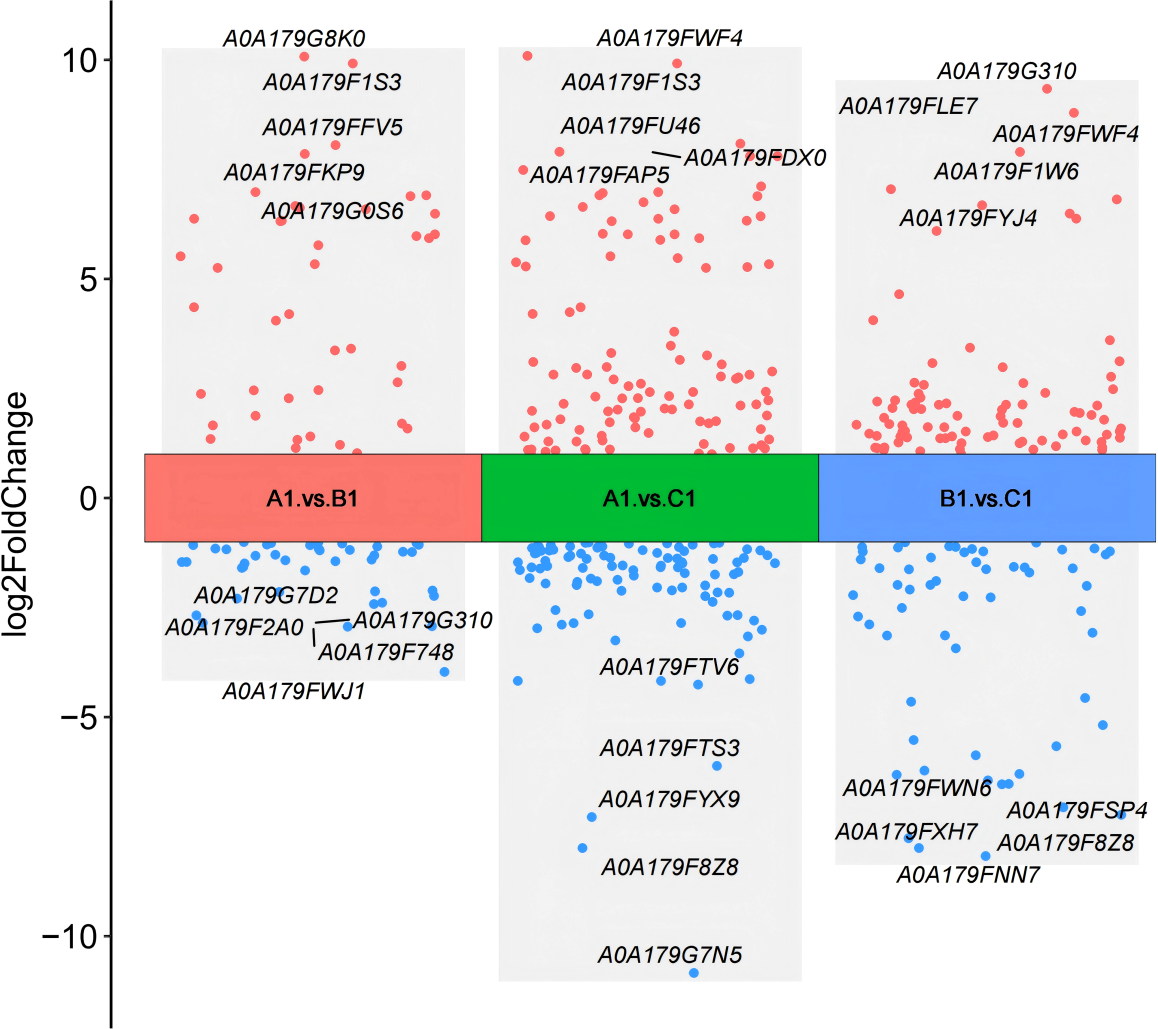
Comparison of volcano plots for differential protein expression between A1 versus B1, A1 versus C1, and B1 versus C1.

Differential protein expression was assessed across three pairwise comparisons: A1 versus B1, A1 versus C1, and B1 versus C1, as detailed in **Table 2**. Volcano plots (**Figure 2**) were employed to visualize the distribution of DEPs. Each plot illustrates the relationship in fold change. Proteins that are significantly upregulated or downregulated are distinctly clustered, with those exhibiting large fold changes and high statistical significance positioned at the extremes of the axes. These visualizations provide a clear method for identifying proteins with the most pronounced expression differences at the three stages (A1, B1, and C1) of *Pochonia chlamydosporia* egg parasitism.

**Table 2:** Summary of differential protein analysis results for A1 versus B1, A1 versus C1, and B1 versus C1.

Comparison group	Total differential proteins	Upregulated proteins	Downregulated proteins
A1 vs. B1	106	51	55
A1 vs. C1	251	123	128
B1 vs. C1	230	136	94

Proteins meeting the rigorous criteria of a fold change greater than 1.5 (either upregulated or downregulated) and exhibiting a *P*-value below 0.05 were designated as significantly DEPs.

**Figure 3:**
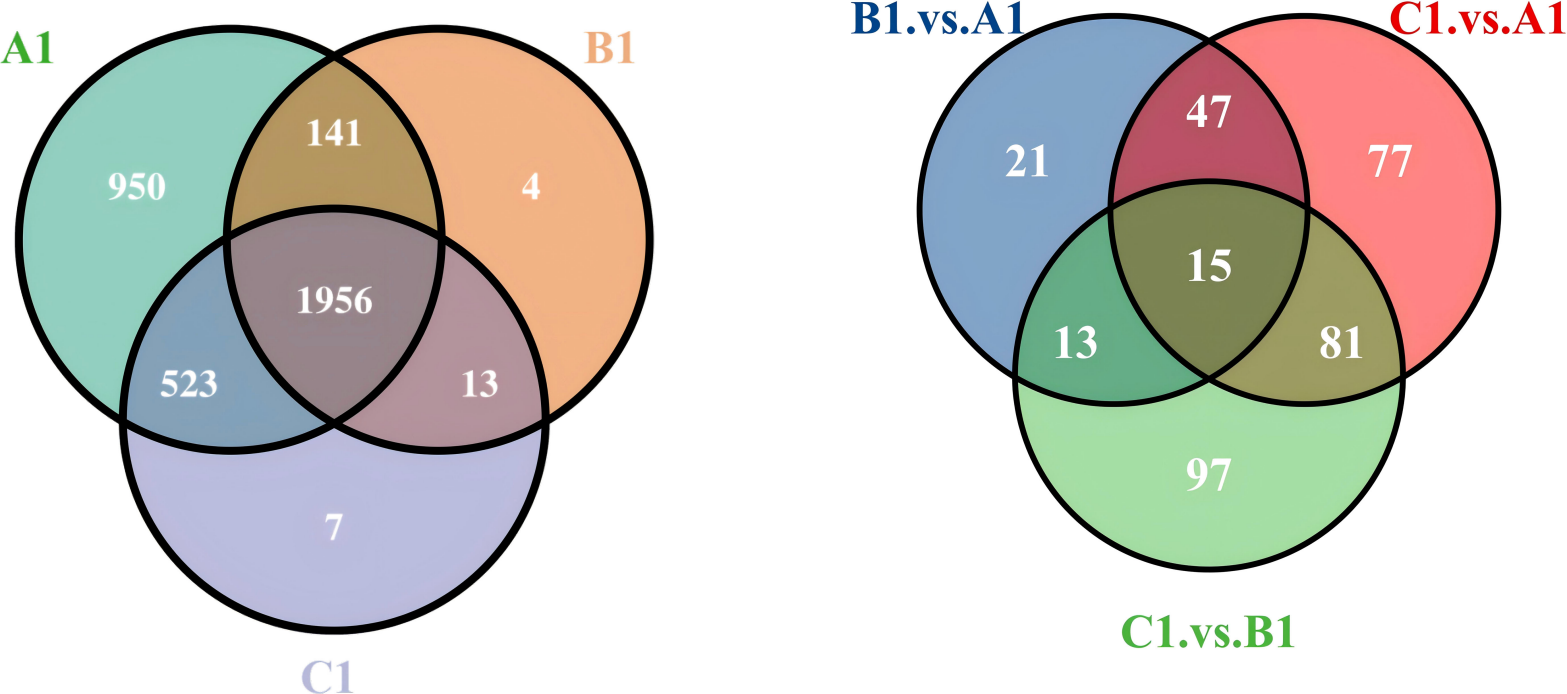
Venn diagram of expressed extracellular proteins at A1/B1/C1 and differential proteins comparisons between A1versus B1, A1 versus C1, and B1 versus C1.

In this study, we used a Venn diagram (**Figure 3**) to analyze the extracellular proteins involved in the fungal hyphal infection of nematode eggs, comparing the DEPs at three stages: early (A1), middle (B1), and late (C1) (Appendix **1**).

The differential protein Venn diagram was generated by comparing the two sets of groups, A1 versus B1, A1 versus C1, and B1 versus C1, to illustrate the shared and unique differential proteins between the groups.

### 3.3 GO functional annotation of differential proteins

**Figure 4:**
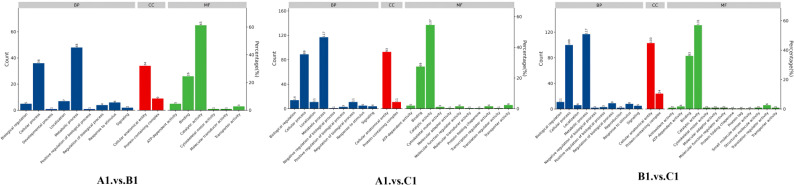
Distribution of differentially abundant proteins according to biological processes (BP), molecular functions (MF), and cellular compartments (CC) in A1 versus B1, A1 versus C1, and B1 versus C1.

The DEPs are significantly involved in several critical biological functions and processes, as indicated by the GO categories (**Figure 4**).

**Figure 5:**
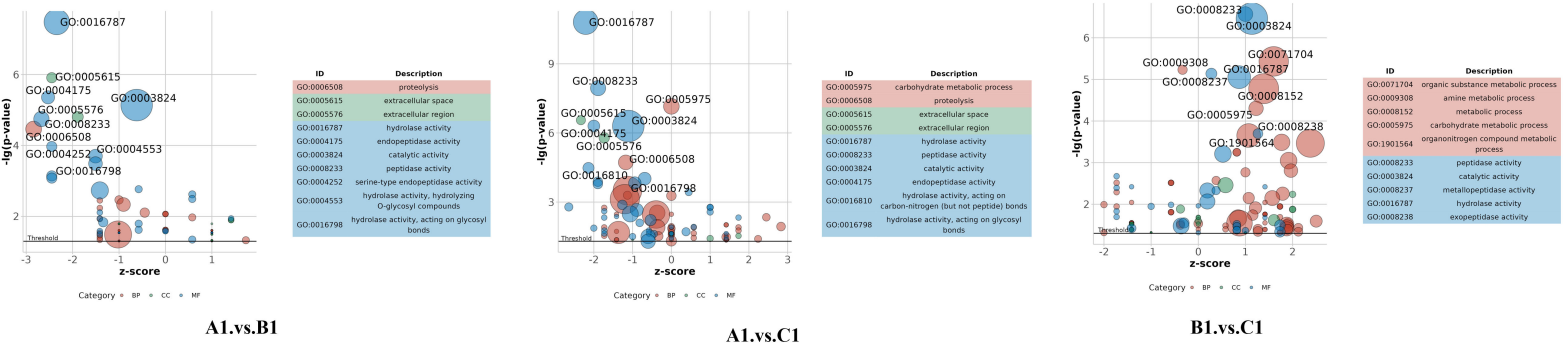
Gene ontology (GO) bubble plot in A1 versus B1, A1 versus C1, and B1 versus C1.

In the GO enrichment analysis of the exoproteins from the A1/B1 group, key enriched GO terms included proteolysis (GO:0006508), hydrolase activity (GO:0016787), and extracellular space (GO:0005615) (**Figure 5**, A1 vs. B1).

The GO enrichment analysis of early- and late-stage exoproteins from the A1/C1 group revealed that enriched terms comprised carbohydrate metabolic process (GO:0005975), proteolysis (GO:0006500), hydrolase activity (GO:0016787), and extracellular space (GO:0005615) (**Figure 5**, A1 vs. C1).

For the B1/C1 group (**Figure 5**, B1 vs. C1), major enrichments included organic substance metabolic process (GO:0071704), carbohydrate metabolic process (GO:0005975), amino acid biosynthetic process (GO:0009308), and biosynthetic process (GO:0008152). Enzyme-related terms focused on peptidase activity (GO:0008233), serine-type peptidase activity (GO:0008237), hydrolase activity (GO:0016787), and enzyme activity (GO:0003824) (**Figure 5**, B1 vs. C1).

### 3.4 KEGG pathway enrichment analysis of the proteins with different abundances

To identify the biological pathway information of the three developmental stages in *Pochonia chlamydosporia*, these proteins were further mapped to the corresponding pathways included in the KEGG database (**Table 3**).

**Table 3:** KEGG pathway analysis of proteins with different abundances in A1, B1, and C1.

KEGG pathway	Pathway ID	Number of proteins
Other glycan degradation	map00511	1
beta-Alanine metabolism	map00410	2
Arginine and proline metabolism	map00330	3
Fatty acid degradation	map00071	3
PPAR signaling pathway	map03320	3
Sphingolipid metabolism	map00600	2
Protein processing in endoplasmic reticulum	map04141	5
Cyanoamino acid metabolism	map00460	3
Valine, leucine, and isoleucine degradation	map00280	6
Tryptophan metabolism	map00380	6

**Figure 6:**
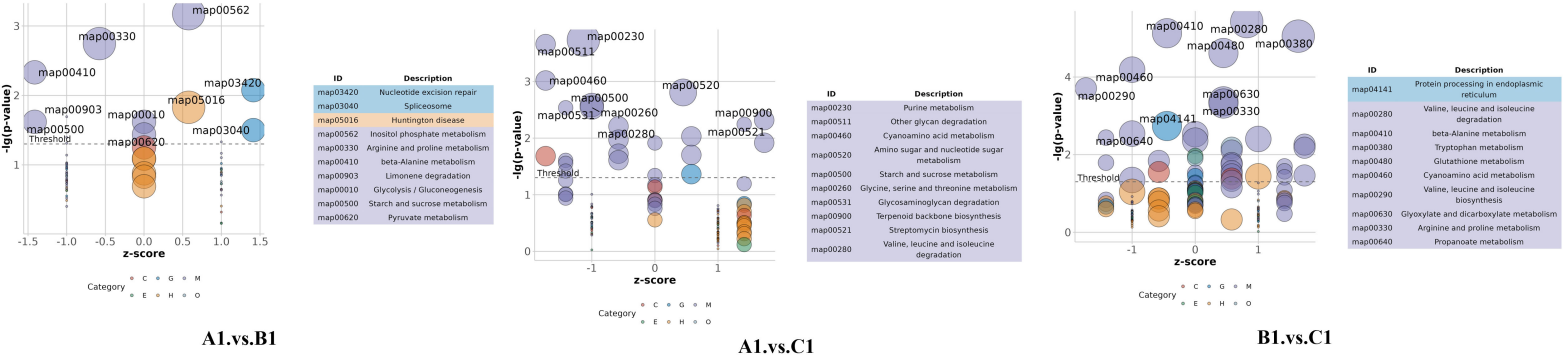
Top 10 enriched KEGG pathways for the comparisons A1 versus B1, A1 versus C1, and B1 versus C1.

**Figure 7:**
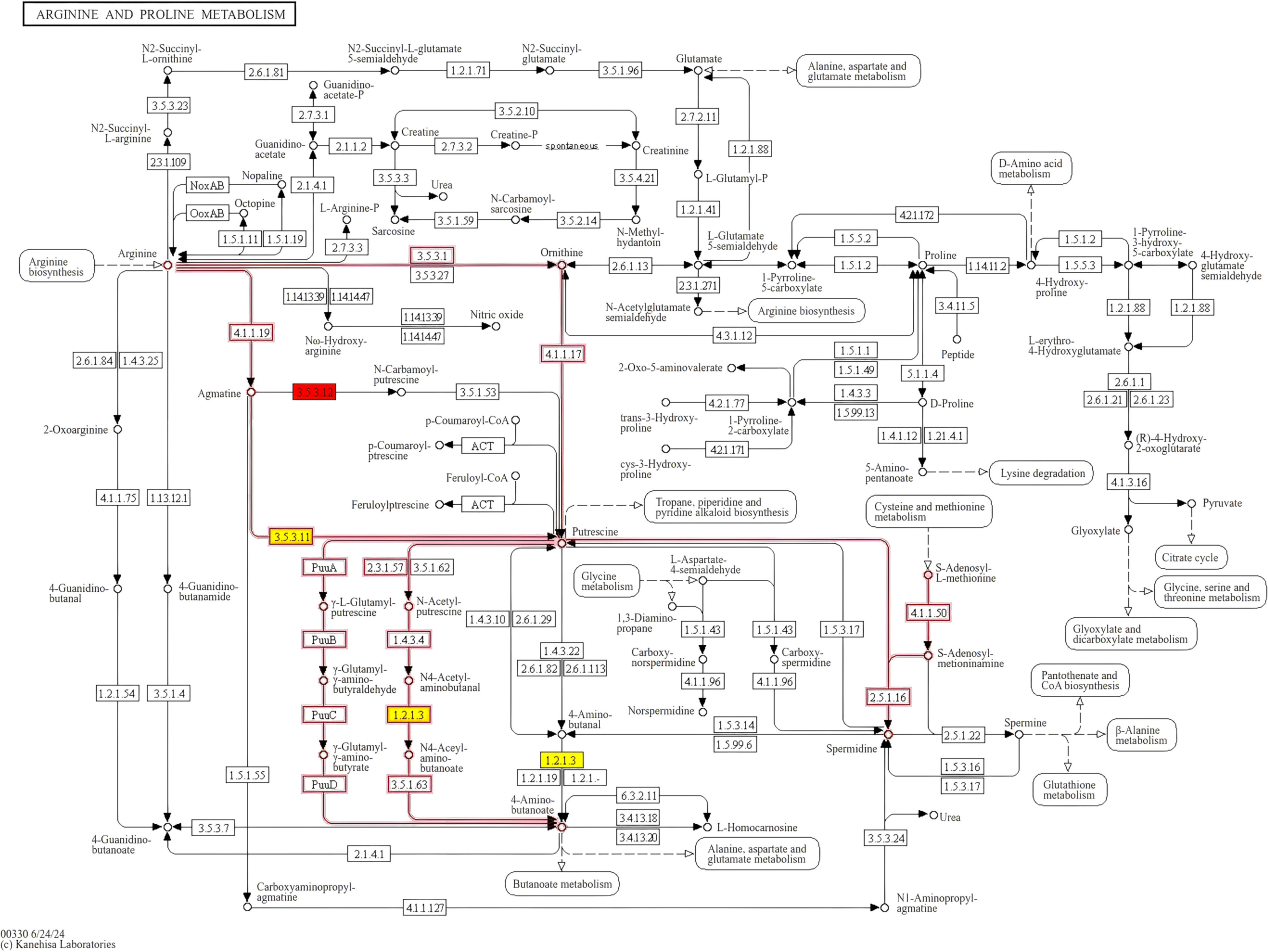
KEGG signaling pathway of arginine and proline metabolism. Proteins labeled in red are significantly upregulated.

The bubble plot shown in **Figure 6** displays the top 10 enriched KEGG pathways for the pairwise comparisons: A1 versus B1, A1 versus C1, and B1 versus C1. The size of each bubble corresponds to the number of differentially expressed genes (DEGs) associated with the pathway, while the color of the bubbles reflects the statistical significance of each pathway. Among these metabolic pathways, a total of nine significantly enriched KEGG pathways were identified, namely, other glycan degradation, beta-alanine metabolism, arginine and proline metabolism, fatty acid degradation, the PPAR signaling pathway, sphingolipid metabolism, cyanoamino acid metabolism, valine, leucine, and isoleucine degradation, and tryptophan metabolism (**Figure 7**).

In the comparison between A1 versus B1 (early vs. mid-stage), the most enriched pathways include map04141: Protein processing in endoplasmic reticulum, map00280: Valine, leucine, and isoleucine degradation, map00410: Beta-alanine metabolism, and map00460: Cyanoamino acid metabolism. These pathways are associated with early cellular stress responses, metabolic shifts, energy metabolism, and metabolic adaptation during infection.

In the A1 versus C1 comparison (early vs. late stage), the most enriched pathways include map03420: Nucleotide excision repair, map03040: Spliceosome, map00903: Glycolysis/Gluconeogenesis, and map00620: Limonene degradation. These pathways are linked to DNA damage repair, RNA processing, energy metabolism, and adaptation to host-specific compounds.

In the B1 versus C1 comparison (mid- vs. late-stage), the most enriched pathways include map00230: Purine metabolism, map00511: Other glycan degradation, map00520: Amino sugar and nucleotide sugar metabolism, and map00521: Streptomycin biosynthesis. These pathways are involved in nucleotide biosynthesis, degradation of host cell wall components, fungal cell wall biosynthesis, and potential antibiotic production.

**Figure 8:**
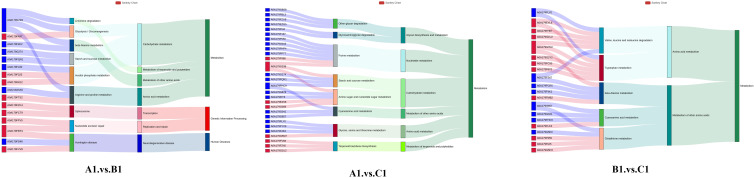
Top 10 gene-to-pathway associations for A1 versus B1, A1 versus C1, B1 versus C1 displayed in a Sankey diagram.

The Sankey plot (**Figure 8**) illustrates the relationship between genes and enriched pathways across different experimental groups (A1 vs. B1, A1 vs. C1, and B1 vs. C1). The directional arrows in the plot represent the association between genes and specific pathways, with the width of the arrows indicating the degree of gene enrichment within these.

### 3.5 Analysis of differential protein abundance at the transcriptional level using qRT-PCR

To compare the changes in protein abundance identified by 4D-DIA related to changes at the gene transcription level, quantitative real-time polymerase chain reaction (qRT-PCR) analysis was performed. Ten proteins were selected for analysis, based on their expression levels identified from the 4D-DIA proteomic data. Among these proteins, three were found to be significantly upregulated, including SNF2 family helicase/ATPase, Histidine phosphotransferase HPT1p, and gamma interferon inducible lysosomal thiol reductase. Conversely, three proteins were identified with lower abundance: metalloprotease, proteinase, alkaline serine protease.

## 4 Discussion

This study conducts a proteomic analysis of *P. chlamydosporia* exoproteins during three stages (A1, B1, C1) of nematode egg infection. Mass spectrometry and bioinformatics reveal stage-specific exoproteomic shifts, uncovering molecular infection mechanisms and identifying key biocontrol-related proteins and pathways.

### 4.1 Microscopic insights into infection morphogenesis

Microscopy observations confirmed that the infection process of *P. chlamydosporia* on *P. equorum* eggs occurs in three distinct phases. These morphological stages are key to understanding the molecular events that drive fungal parasitism and infection progression (Dai et al., 2017).

In comparison to previous studies on *P. chlamydosporia* or other fungal nematode biocontrol agents like *Duddingtonia flagrans* (Dudd.) R.C. Cooke (Ascomycota, Orbiliomycetes, Orbiliales, Orbiliaceae, *Arthrobotrys*) and *Monacrosporium thaumasium* (Drechsler) Subram (Ascomycota, Sordariomycetes, Pezizomycotina, Orbiliaceae, *Arthrobotrys*), which also undergo similar infection stages, our observations of hyphal penetration and egg breakdown align with the findings of Silva et al. (2011) (Silva et al., 2011), who noted that Pochonia species exhibit similar morphological patterns during early and mid-stages of nematode egg infection. However, the resolution of our TEM data provides a finer level of detail, revealing subcellular dynamics during fungal invasion, which was less emphasized in earlier studies. Specifically, we observed that the initial hyphal growth forms specialized attachment cells, which anchor the fungus to the egg surface. Following this, the fungus produces infection structures resembling “infection peg-like” structures, which have a pointed tip that pierces the eggshell. This penetration process, while mechanically facilitated, also involves enzymatic degradation and possible chemical digestion of the egg’s outer layers. Notably, TEM observations revealed that the eggshell undergoes significant deformation, with visible wrinkling and shrinking, likely due to both the mechanical action of the infection peg and enzymatic or chemical digestion. This detailed observation of eggshell breakdown offers a unique contribution to the understanding of the fungal parasitism process and extends the findings of previous studies, such as those by Avelar Monteiro et al. (2017) (Avelar Monteiro et al., 2017), by highlighting the dual role of mechanical and biochemical mechanisms during hyphal penetration.

### 4.2 Temporal dynamics of exoproteomic responses during infection stages

In the early stage, proteins related to adhesion, immune evasion, and genome stability play a crucial role in facilitating fungal establishment. The upregulation of fungal-specific transcription factors, such as VFPPC_15959, highlights the fungus’s ability to finely tune its transcriptional responses to the host’s immune environment, thus activating genes associated with pathogenicity and immune evasion. Similarly, previous studies have shown that during the pathogenesis of nematode eggs, *P. chlamydosporia* expresses transcription factors belonging to the Zn2Cys6 fungal-type, bZIP, and bromodomain-containing TF families, which are abundantly represented in the *P. chlamydosporia* genome (Rosso et al., 2011). VFPPC_13466 (histidine phosphotransferase HPT1p), a protein kinase, plays a crucial role in signal transduction. This class of proteins has been implicated in regulating virulence genes in the entomopathogenic fungus *Metarhizium. anisopliae* (Metschn.) Sorokin (Ascomycota; Pezizomycotina; Sordariomycetes; Hypocreales; Clavicipitaceae; *Metarhizium*) (Fang et al., 2009), allowing the fungus to manage its growth and effectively adapt to immune challenges. These proteomic changes highlight the fungus’s multi-layered strategies—transcriptional regulation, immune evasion, and signal transduction—which collectively facilitate its successful establishment and survival in the host during the early stages of infection. During early infection, *P. chlamydosporia* secretes a variety of hydrolases and proteases into the extracellular space. These enzymes are pivotal for breaking down the host’s physical barriers, including the eggshell and surrounding cellular matrix. The term proteolysis (GO:0006508) points directly to the fungal ability to degrade host proteins, a fundamental mechanism in fungal pathogenesis. As discussed by Lin et al. (2018), secreted proteins involved in responses to nutrient stress are mainly comprised of proteases and glycoside hydrolases (Lin et al., 2018). Additionally, hydrolase activity (GO:0016787) suggests that the fungal enzymes are versatile, degrading a broad range of substrates, including proteins and polysaccharides. This broad spectrum of enzymatic activity facilitates both host invasion and immunity.

In the middle stage, enzymes involved in tissue degradation and immune suppression enable the fungus to invade deeper tissues and overcome host defenses. The exoproteome exhibits a significant increase in proteins associated with the degradation of host tissues and immune defenses. The tough outer shell of nematode eggs constitutes a physical barrier to fungal hyphal invasion, and its main components include proteins, chitin, and various polysaccharides (Bird and Mcclure, 1976; Luyao et al., 2023). The upregulation of endo-β-1,6-galactanase enables the enzyme to specifically target the galactan-mannan structures in the eggshell. By catalyzing hydrolysis, it disrupts the eggshell’s structural integrity, creating a pathway for hyphal penetration. Similar mechanisms have been reported in *Arthrobotrys oligospora* (Fresen.) (Ascomycota; Pezizomycotina; Orbiliomycetes; Orbiliales; Orbiliaceae; *Orbilia*) and *D. flagrans* (Liang et al., 2019; Yang et al., 2011), is likely to have the same mechanism of action, so these changes may be related to the early identification and adhesion of nematodes to predatory fungi. Proteinase secretion aids in immune evasion by degrading host immune proteins and extracellular matrix components, facilitating deeper tissue invasion, as described by Aranda-Martinez et al. (2016) and Huang et al. (2004) (Aranda-Martinez et al., 2016; Huang et al., 2004). Furthermore, the secretion of chitinase supports the breakdown of host structures, aligning with findings by Morton et al. (2003) and Tikhonov et al. (2002), who demonstrated the critical role of proteases and chitinases in enhancing fungal invasion (Morton et al., 2003; Tikhonov et al., 2002). These observations collectively suggest a shift towards active tissue degradation and immune suppression, key strategies that foster fungal growth and parasitism during this stage. As fungal infection progresses from the mid (B1) to the late (C1) stages, the fungus undergoes significant metabolic shifts in response to changing host conditions. However, as the infection advances and host nutrients deplete, the fungus reduces these metabolic processes, shifting towards a more energy-conserving state focused on survival and reproduction. This transition is marked by decreased carbohydrate and amino acid metabolism, protein degradation, and enzyme activity in the late infection stage. Similarly, Liang et al. (2019) indicated that as most of the nematodes were captured and digested, external stimuli were reduced, leading to a gradual attenuation of the signaling pathways in the nematode-trapping fungus *D. flagrans* (Liang et al., 2019).

In the late stage, proteins regulating nutrient exploitation and fungal growth ensure continued proliferation and survival within the host. The exoproteome is predominantly characterized by proteins involved in nutrient exploitation from the host and the regulation of fungal growth. Notably, the upregulation of VFPPC_04753 (aminoalcoholphosphotransferase: AAPTs) implies that *P. chlamydosporia* may enhance its capacity for cell membrane repair and synthesis, which is crucial for maintaining cell integrity under the stress imposed by the host’s immune response. AAPTs are integral membrane proteins whose activity has been shown to occur in microsomal fractions of animal, yeast, and plant cells (Bai et al., 2021; Liu et al., 2015). These enzymes play a pivotal role in the synthesis of essential membrane components, providing a potential mechanism for the fungus to adapt to the hostile environment of the host. This supports the idea that *P. chlamydosporia* upregulates AAPTs to facilitate membrane stability and function, essential for its survival and parasitic interactions. In the later stage of hyphal infection of nematode eggs, the interaction between the hyphae and nematode eggs becomes closer and more complex. This protein may be involved in the regulation of intracellular redox balance, which is crucial for the survival of hyphae under oxidative stress. During the infection process, the nematode eggs may initiate defense mechanisms to produce some substances with oxidative activity. The upregulation of this protein helps the hyphae to scavenge excessive reactive oxygen species (ROS), protecting intracellular biomacromolecules from oxidative damage. As noted in previous studies, plants are known to produce ROS as a defense mechanism against pathogen invasion (Camejo et al., 2019), and Morano et al. (2012) demonstrated that antioxidant enzymes can protect cells from oxidative damage caused by ROS (Morano et al., 2012). This finding can be used to support the role of antioxidant enzymes in defending against oxidative damage. At the early stages, the fungal focus on proteolysis facilitates host invasion, whereas in the late stages, the focus shifts toward utilizing the host’s carbohydrate reserves. This transition indicates a metabolic adaptation essential for fungal survival and growth within the nematode egg. Ramesh et al. (2016) also observed a similar metabolic shift in *P. chlamydosporia*, where the fungus transitions from proteolysis to nutrient acquisition, particularly sugars and amino acids, to support its growth during infection stages (Ramesh et al., 2016). In addition to carbohydrate metabolism, ribonuclease activity (GO:0016810) emerges as an enriched term in the late infection stages, pointing to a strategy where *P. chlamydosporia* may manipulate host RNA metabolism. This is crucial for suppressing the host immune response or altering cellular processes that could hinder fungal proliferation. As Olombrada et al. (2017) suggested, ribonucleases may degrade host RNA and interfere with immune signaling, providing *P. chlamydosporia* a competitive advantage by preventing host defense mechanisms from being fully activated (Olombrada et al., 2017). Furthermore, the sustained enrichment of proteolysis in the late stages suggests that protein degradation remains an essential process for maintaining fungal growth within the host (Gortari and Hours, 2008; Hirano et al., 2008; Shinya et al., 2008).

### 4.3 Key KEGG pathway analysis of the proteins with different abundance

KEGG analysis reveals that during the early stage of infection, the mitogen-activated protein kinase (MAPK) signaling pathway exhibits significant dynamic changes, with notable upregulation of transcriptional repressors and histidine phosphotransferases. Although direct evidence demonstrating the effective suppression of host immune responses by these factors is currently lacking, this upregulation is highly likely associated with the fungus establishing a survival niche and evading host immune surveillance. The MAPK signaling pathway has long been recognized as a key player in the infection process of plant pathogenic fungi. Hamel et al. (2012) systematically summarized the role of this pathway in plant pathogenic fungi, indicating that the invasive growth pathway (IG pathway) regulates infection-related morphogenesis, such as the formation of appressoria, infection pegs, and invasive hyphae, thereby strongly supporting fungal infection (Hamel et al., 2012). Concurrently, histidine phosphotransferase plays a critical role in enhancing fungal infectivity. It creates a more favorable infection environment by modulating the metabolism and immune responses of host cells. Studies on *Magnaporthe oryzae* by Zhao et al. (2005) showed that the histidine phosphotransferase deletion mutant (Δhpt) exhibited significantly reduced virulence and impaired infection ability, accompanied by excessive activation of host immune responses (Zhao et al., 2005). The upregulation of histidine phosphotransferase observed in this study with *P. chlamydosporia* aligns with these findings, confirming its key role in fungal growth and infection. Additionally, research by Van Thuat et al. (2012) and Jain et al. (2011) has also demonstrated the important role of the MAPK pathway in other plant pathogenic fungi. FgOS-2 (a MAPK gene in *Fusarium graminearum*) and MpkA (a MAPK gene in *Aspergillus nidulans*) are similarly decisive in regulating cell wall integrity, oxidative stress responses, and pathogenicity (Jain et al., 2011; Van Thuat et al., 2012). These studies indicate that the MAPK pathway is indispensable for both cell wall structural remodeling and countering host immune responses.

During the mid-infection stage, arginine and proline metabolic pathways also play pivotal roles. Notably, peptidyl arginine deiminase-like enzymes are downregulated, while aldehyde dehydrogenase (NAD^+^) and arginine enzyme family domain-containing proteins show upregulation trends. Arginine serves not only as a key precursor for protein synthesis but also participates in fungal virulence regulation through the urea cycle and nitric oxide synthesis pathway (Wu and Morris, 1998). Proline, meanwhile, aids fungi in coping with environmental stresses during infection by maintaining cellular osmotic pressure and antioxidant capacity (Lehmann et al., 2010). Studies on the interaction between *Trichoderma harzianum* and plants have revealed that arginine and proline metabolic pathways are upregulated during symbiosis, enabling fungi to adapt to the plant root environment and promote plant growth (Shoresh et al., 2010).

The upregulation of sphingolipid metabolism in the mid- to late stages suggests intricate interactions between *P. chlamydosporia* and its host. Sphingolipids like ceramide and sphingosine are crucial for regulating immune responses, cell survival, and apoptosis. While the host may upregulate sphingolipid metabolism as a defensive measure, *P. chlamydosporia* may modulate this pathway to trigger host cell apoptosis or autophagy, enhancing its ability to evade immune detection. Previous research by Chiang et al. (2014) has shown that sphingolipids and their metabolite neutral amides have a wide distribution in the cell membranes of animal and plant tissues (Chiang et al., 2014). They are actively involved in numerous crucial signal transduction processes like cell growth, differentiation, the aging process, and programmed cell death, thereby fulfilling various biological functions within cells. With regard to nematodes, these substances are capable of influencing the nematode’s nervous system and are essential for the development of the epidermal stratum corneum. These findings are consistent with those reported by Liang et al. (2019) (Liang et al., 2019). They showed that during the early stages of infection, the upregulation of sphingomyelin phosphodiesterase and downregulation of sphinganine C4-monooxygenase and GTPase Kras accelerated sphingolipid metabolism, leading to an increase in neutral amide content. Between late stages, neutral ceramides were upregulated, further promoting sphingolipid metabolism. Additionally, this metabolic shift may provide energy for the fungus, helping it adapt to nutritional stress and hypoxic conditions within the host.

The functional roles of DEPs were validated through qRT-PCR analysis, which confirmed the trends observed in the proteomic data. The upregulation of SNF2 family helicase and HPT1p (a phosphotransferase) during early stages suggests involvement in DNA repair and host cell signaling modulation. The downregulation of metalloproteases and alkaline serine proteases at later stages highlights the intricate regulation of protein degradation, reflecting the need for precise control over host tissue breakdown and fungal growth. These results support the hypothesis that fungal infection is a highly regulated process, with specific proteins being upregulated or downregulated in response to different stages of infection.

## 5 Conclusion

This study provides a comprehensive investigation of *P. chlamydosporia* infection on nematode eggs, combining advanced microscopy and proteomics to uncover the molecular mechanisms of parasitism. For the first time, we visualized the ultrastructural details of the interaction using TEM and identified significant changes in protein expression across different infection stages. Key pathways involved in proteolysis and amino acid metabolism were highlighted, offering new insights into the fungal parasitic strategy. These findings not only deepen our understanding of the host-colonization mechanisms but also hold significant implications for the development of *P. chlamydosporia* as a sustainable biological control agent. The molecular strategies identified here could be leveraged to optimize their application in integrated animal-parasitic helminth management systems, and future research should focus on translating these insights into practical solutions for large-scale agricultural use.

## Data Availability

The original contributions presented in the study are publicly available. This data can be found here: ProteomeXchange Consortium / PXD067347.
